# Advances of nanomaterials‐based strategies for fighting against COVID‐19

**DOI:** 10.1002/VIW.20200180

**Published:** 2021-05-05

**Authors:** Chunxi Zeng, Xucheng Hou, Margaret Bohmer, Yizhou Dong

**Affiliations:** ^1^ Division of Pharmaceutics & Pharmacology, College of Pharmacy The Ohio State University Columbus Ohio USA; ^2^ College of Medicine The Ohio State University Columbus Ohio USA; ^3^ Department Biomedical Engineering The Ohio State University Columbus Ohio USA; ^4^ The Center for Clinical and Translational Science The Ohio State University Columbus Ohio USA; ^5^ The Comprehensive Cancer Center The Ohio State University Columbus Ohio USA; ^6^ Dorothy M. Davis Heart & Lung Research Institute The Ohio State University Columbus Ohio USA; ^7^ Department of Radiation Oncology The Ohio State University Columbus Ohio USA

**Keywords:** COVID‐19, diagnostics, nanomaterials, SARS‐CoV‐2, therapeutics, vaccines

## Abstract

The severe acute respiratory syndrome coronavirus 2 (SARS‐CoV‐2) has infected over 100 million people globally due to its high infectivity. After decades of efforts on the studies of nanomaterials, researchers have applied nanomaterials‐based strategies to combat the pandemic of the coronavirus disease 2019 (COVID‐19). First, nanomaterials facilitate the development of easy, fast, and low‐cost diagnostic assays to detect SARS‐CoV‐2 and related biomarkers. Second, nanomaterials enable the efficient delivery of viral antigens to antigen‐presenting cells or serve as adjuvants in the host, leading to vaccine development at an unprecedented pace. Lastly, nanomaterials‐based treatments may inhibit SARS‐CoV‐2 replication and reduce inflammation. Overall, nanomaterials have played important roles in controlling this COVID‐19 pandemic. Here, we provide a brief overview of the representative examples of nanomaterials‐based diagnostics, vaccines, and therapeutics in the fight against COVID‐19.

## Graphical abstract

The COVID‐19 pandemic has infected millions and millions of people worldwide. This mini‐review summarizes the nanomaterial‐enabled applications for COVID‐19 diagnostics, vaccines, and therapeutics.

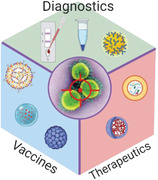

## 1 INTRODUCTION

The severe acute respiratory syndrome coronavirus 2 (SARS‐CoV‐2) has already infected more than one hundred million people worldwide, leading to the death of over two million patients as of February 15, 2021.^[^ ^1^ ^]^ The numbers of infection and fatality continue to rise every day amid this COVID‐19 pandemic.^[^ ^1^ ^]^ The structural features of the viral particle are of great importance for understanding the process of SARS‐CoV‐2 infections and developing effective countermeasures. Researchers have found that the SARS‐CoV‐2 genome is made of a single‐stranded RNA.^[^ ^2^ ^]^ Its viral particle contains four structural proteins, including: (1) spike (S) protein with a receptor‐binding domain (RBD) that enables the entry of SARS‐CoV‐2 into host cells and a heptad repeat (HR) domain that is conserved among many human coronaviruses; (2) membrane (M) protein that stabilizes the viral membrane ; (3) envelope (E) protein that contributes to viral budding; and (4) nucleocapsid (N) protein that packages the genomic RNA of the virus.^[^ ^2^, ^3^, ^4^ ^]^

**FIGURE 1:**
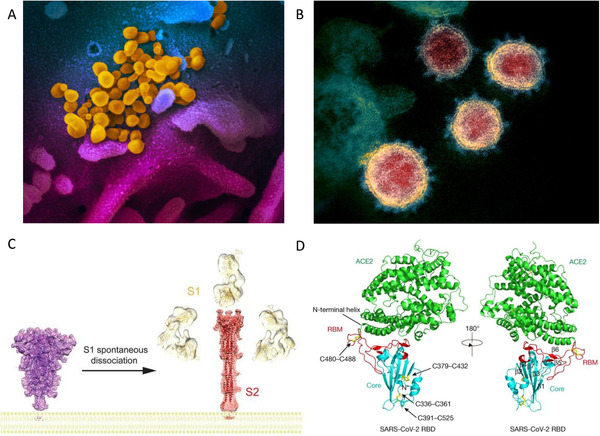
Visualization of SARS‐CoV‐2. Scanning electron microscopy image (A) and transmission electron microscopy image (B) of SARS‐CoV‐2 that was isolated from COVID‐19 patients and was cultured with cells. Images were color enhanced. Credit: NIAID.^[^ ^19^ ^]^ (C) Spike protein in prefusion and postfusion states determined by Cryo‐EM (reprint with permission.^[^ ^9^ ^]^ Copyright 2020 AAAS). (D) The interactions between spike and human ACE2 determined by X‐ray crystallography (reprint with permission.^[^ ^10^ ^]^ Copyright 2020 Springer Nature)

To visualize the morphology of the SARS‐CoV‐2 viral particle, advanced imaging technologies such as scanning electron microscopy (SEM) and transmission electron microscopy (TEM) were utilized.^[^ ^5^ ^]^ SEM revealed the exterior 3D shape of SARS‐CoV‐2 by detecting the interactions of the electrons with the sample surface (Figure 1A). Meanwhile, TEM generated a 2D thin layer image of SARS‐CoV‐2 by sensing the electrons transmitted through the viral sample (Figure 1B). These images indicated that SARS‐CoV‐2 particles had an average diameter of ∼100 nm with spike proteins of 9–12 nm protruding from the surface of viral particles.^[^ ^6^, ^7^, ^8^ ^]^ To further elucidate the structural information of the spike protein, two additional technologies were employed: cryo‐electron microscopy (Cryo‐EM) and X‐ray crystallography. Cryo‐EM was utilized to study the structure of spike protein at its prefusion and postfusion states during its interaction with angiotensin‐converting enzyme 2 (ACE2), the cell surface receptor of SARS‐CoV‐2 (Figure 1C).^[^ ^9^ ^]^ X‐ray crystallography pinpointed the amino acid residues responsible for the spike and ACE2 interactions (Figure 1D).^[^ ^10^ ^]^ Results from these studies revealed that the binding affinity of SARS‐CoV‐2 to human ACE2 (hACE2) was higher than that of SARS‐CoV. Moreover, a mutation in the spike protein (D614G) increased the infectivity of SARS‐CoV‐2.^[^ ^11^ ^]^ Such a mutation increased viral loads in the infected COVID‐19 patients, while no obvious increase in disease severity was reported.^[^ ^11^ ^]^

To control the rapid spread of SARS‐CoV‐2, tremendous efforts are being exerted to accelerate the development of diagnostics, vaccines, and treatments.^[^ ^12^ ^]^ Previous studies on nanomaterials have created valuable knowledge that is being employed for overcoming the COVID‐19 pandemic. Regarding disease diagnostics, nanomaterials enable sensitive, quick, and convenient detection methods. Recently, some colloidal gold nanoparticles‐based serology point‐of‐care tests have received Emergency Use Authorization (EUA) from the US Food and Drug Administration (FDA).^[^ ^13^ ^]^ This facilitates the fast identification of individuals who may have asymptomatic or past SARS‐CoV‐2 infection. Furthermore, nanomaterials can help protect antigen components until they are delivered to the antigen‐presenting cells, thus expediting vaccine development. Particularly, mRNA vaccines delivered via lipid nanoparticles obtained regulatory authorization at an unprecedented speed. Recent clinical results revealed that vaccine candidates, mRNA‐1273 and BNT162b2, triggered strong cell responses (CD 8+ CTLs and CD4+ T helper cells) and high antibody levels against SARS‐CoV‐2.^[^ ^14^, ^15^, ^16^ ^]^ Finally, nanomaterials, with the ability to improve the pharmacokinetic and pharmacodynamic aspects of drugs, can minimize the side effects of drugs.^[^ ^17^ ^]^ Moreover, stem cell‐ or T cell‐derived exosomes may function as independent therapeutics, which have shown therapeutic potentials for SARS‐CoV‐2 in clinical trials (NCT04493242, NCT04276987, NCT04389385).^[^ ^18^ ^]^

Herein, we briefly review the representative examples of nanomaterials‐based diagnostics, vaccines, and therapeutics for SARS‐CoV‐2 (Table 1). We also describe their features and clinical status. Our overarching goal throughout this article is to highlight the versatile potentials of nanomaterials in combating the COVID‐19 pandemic.

**TABLE 1:** Representative nanomaterials for COVID‐19 diagnostics, vaccines, and therapeutics

Diagnostics				
Core nanomaterials	Application	Key features	Clinical stage^a^	Ref.
Colloids gold‐nanoparticles (NPs) conjugated to RBD	Detect anti‐RBD IgG and IgM in blood on a lateral flow strip	Rapid test within 15 minutes; readout by naked eyes.	Pre	[20]
Colloids gold‐NPs	Detect antinucleocapsid IgM in blood on a lateral flow strip	Rapid test within 15 minutes; readout by naked eyes.	Pre	[21]
Gold‐NPs conjugated to hACE2	Detect viral particle in serum samples	Rapid test within 15 minutes; readout using a microplate reader or smartphone‐connected device.	Pre	[22]
Gold‐NPs conjugated to antisense oligonucleotides	Detect viral RNA in swab samples in test tube	Rapid test within 10 minutes after viral RNA extraction; readout by naked eyes in test tubes.	Pre	[23]
Gold‐NPs conjugated to antisense oligonucleotides and a graphene layer	Detect viral RNA in nasal swab or saliva samples	Rapid test with 5‐minute incubation after viral RNA extraction; detect electrochemical signal on a biosensor chip.	Pre	[24]
Gold nanoisland on a chip	Detect viral RNA on a chip sensor	Lowest detection limit: 0.22 pM viral RNA; readout using a plasmonic sensing system.	Pre	[25]
Gold NPs linked to organic ligands	Detect COVID‐19 or other conditions from exhaled breath	Organic ligands array mediates change of electric resistance; detection is based on machine learning of signal pattern.	Pre	[26]
Selenium NPs	Detect anti‐nucleoprotein IgG and IgM in blood on a lateral flow strip	Rapid test within 10 minutes; readout by naked eyes.	Pre	[27]
Polystyrene‐NPs	Detect anti‐nucleoprotein IgG in blood on a lateral flow test strip	Rapid test within 10 minutes; readout using a portable fluorescence reader.	Pre	[28]
Enzyme‐functionalized NPs containing iron and cobalt	Detect RBD antigen on a lateral flow strip	Rapid test within 16 minutes; readout using a smartphone camera.	Pre	[29]
Streptavidin‐dye‐coated polymer NPs	Detect viral RNA on a lateral flow strip	Rapid test within 1 hour; constant reaction temperature; compatible with various clinical samples.	Pre	[30]
Cobalt‐functionalized TiO2 nanotubes	Detect RBD antigen on an electrochemical biosensor	Electrochemical signal‐based detection without using immobilized antibody; rapid detection within 30 seconds.	Pre	[31]
Graphene sheet	Detect spike protein in the swab samples on a chip sensor	Readout using a field‐effect transistor‐based biosensing device.	Pre	[32]
Nanoflakes of reduced‐graphene‐oxide	Detect anti‐spike and anti‐RBD antibodies on a 3D‐printed test chip	Spike and RBD are immobilized on the nanoflakes, which are connected to gold electrodes; the test chip can be regenerated and reused; readout using a smartphone.	Pre	[33]
Graphene electrodes	Detect one antigen, two antibodies and one disease biomarker simultaneously in a handheld device.	Multiplexing detection yields more information for diagnosis; compatible with blood and saliva samples; readout using a smartphone.	Pre	[34]
Magnetic NPs functionalized with streptavidin	Detect anti‐spike IgG in blood via a filtration column	Readout using a portable magnetic reader.	Pre	[35]
NPs with a magnetic core and a gold plasmonic shell	Detect viral RNA using plasmonic RT‐PCR	Rapid test within 17 minutes; PCR amplification and fluorescence detection in one portable device.	Pre	[36]
Polymer NPs	Detect viral RNA on a lateral flow strip after RT‐LAMP amplification	Whole process takes 1 hour; readout by naked eyes.	Pre	[37]
Iron‐containing magnetic NPs	Extract viral RNA efficiently for subsequent RT‐PCR	Extract viral RNA within 20 minutes.	Pre	[38]
DNA‐based nanoswitch	Detect viral RNA by gel mobility shift	Binding of nanoswitch to fragmented viral RNA induced conformational change.	Pre	[39]
**Vaccines**				
Lipid nanoparticles (LNPs)	Deliver an mRNA vaccine (BNT162b2)	mRNA encodes prefusion stabilized spike; stored at –80°C and stable at 4°C for 4 days.	EUA	[40] NCT04380701
Ionizable LNPs	Deliver an mRNA vaccine (mRNA‐1273)	mRNA encodes prefusion stabilized spike; stored at –20°C and stable at 4°C for 30 days.	EUA	[41] NCT04470427
Ionizable LNPs	Deliver an mRNA vaccine (CVnCoV)	mRNA encodes a prefusion‐stabilized spike.	III	[42] NCT04652102
Ionizable LNPs	Deliver an mRNA vaccine (ARCoV)	mRNA encodes an RBD fragment. Formulation is stable at 25°C for 7 days.	I	[43] ChiCTR2000034112
Ionizable LNPs	Deliver an mRNA vaccine (MRT5500)	mRNA encodes a prefusion‐stabilized spike with furin cleavage site mutation.	Pre	[44]
TT3 LNPs	Deliver a NASAR mRNA vaccine	mRNA utilizes the optimized NASAR UTRs for strong antigen expression.	Pre	[45]
MC3 LNPs	Deliver a three‐mRNA cocktail vaccine	mRNA encodes virus‐like particles to induce immunity.	Pre	[46]
Ionizable LNPs	Deliver a self‐amplifying mRNA vaccine (LNP‐nCoVsaRNA)	SA‐mRNA encodes a prefusion‐stabilized spike.	I	[47] ISRCTN17072692
Ionizable LNPs	Deliver a self‐amplifying mRNA vaccine (LUNAR‐COV19)	SA‐mRNA encodes a prefusion‐stabilized spike.	II	[48] NCT04728347
Liposomes	Deliver a recombinant trimeric spike as vaccine	Single‐dose intranasal vaccination; induced mucosal IgA production in lung and nasal compartment.	Pre	[49]
Liposomes with cobaltporphyrin‐phospholipid	Deliver a recombinant RBD as vaccine	The liposome forms particulates with RBD.	Pre	[50]
Squalene‐based cationic nanoemulsion	Deliver a self‐amplifying mRNA vaccine	SA‐mRNA encodes the spike protein; squalene provides adjuvant activity.	Pre	[51]
Saponin‐based nanoemulsion named Matrix‐M1	Provide adjuvant activity for a recombinant spike protein vaccine.	Matrix M1 is a mixture of two saponin‐based fractions to balance adjuvant activities and side effects.	III	[52] NCT04583995 NCT04368988
Squalene‐based nanoemulsion named MF59	Provide adjuvant activity for a recombinant spike protein vaccine (V451).	MF59 is an FDA‐approved adjuvant for an influenza vaccine.	I	[53]
Particulate alum‐stabilized pickering emulsion (PAPE)	Provide adjuvant activity for a recombinant RBD vaccine.	Enhances antigen uptake and presentation.	Pre	[54]
Self‐assembled protein NPs with I53‐50 core	Present recombinant RBD on the surface of protein NPs as a vaccine.	RBD is genetically fused to one protein component.	Pre	[55]
Self‐assembled protein NPs with I53‐50 core	Present stabilized recombinant spike on the surface of protein NPs as a vaccine.	Spike is genetically fused to one protein component.	Pre	[56]
Self‐assembled protein NPs with lumazine synthase (LuS) core	Present recombinant spike on the surface of protein NPs as a vaccine.	Spike is linked to NPs via SpyTag:SpyCatcher.	Pre	[57]
Self‐assembled protein NPs with ferritin core	Present recombinant RBD and/or heptad repeat (HR) subunits of the spike on the surface of protein NPs as a vaccine.	Spike is linked to NPs via SpyTag:SpyCatcher. HR may induce cross reactivity against other coronaviruses.	Pre	[58]
Self‐assembled protein NPs with three different cores (ferritin, E2p, or I3‐01v9)	Present mutated recombinant spike on the surface of protein NPs as vaccines.	Spike is linked to NPs via SpyTag:SpyCatcher. In the recombinant spike, two amino acids were mutated to glycine and HR2 domain was removed.	Pre	[59]
Self‐assembled protein NPs with virus‐like particle core	Present multiple distinct RBDs as a vaccine	RBDs are linked to NPs via SpyTag: SpyCatcher; diverse RBDs induced cross‐reactivity against different coronaviruses.	Pre	[60]
**Therapeutics**				
Thin shell polymer	Encapsulate catalase to degrade ROS	Thin shell protects catalase while permitting ROS transport; administered by Nebulizations or intravenous injection.	Pre	[61]
Polydopamine‐poly(ethylene glycol) nanoparticulates	Encapsulate DNase‐1 to degrade cell‐free DNA	Suppress neutrophil activities and the cytokine storm; administered by intravenous injection.	Pre	[62]
MC3 LNPs	Inhibit infection with hACE2 expressed from an mRNA.	Administered by intravenous injection or intratracheal instillation.	Pre	[63]
Cellular nanosponges	Inhibit infection with hACE2 available on cell membrane.	The scaffold is made of polymer nanoparticles.	Pre	[64]
Nanorods coated with cell membrane	Inhibit infection with hACE2 available on cell membrane.	The scaffold is made of mesoporous silica‐coated bismuth nanorods.	Pre	[65]
Stem cell‐derived exosome named ExoFlo	Treat hospitalized COVID‐19 patients.	Administered by intravenous infusion.	II	[18] NCT04493242
Stem cell‐derived exosome	Treat severe COVID‐19 patients.	Administered by aerosol inhalation.	I	NCT04276987
T cell‐derived exosome	Treat early stage COVID‐19.	SARS‐CoV‐2 peptides and cytokines are used to facilitate exosome secretion from T cells.	I/II	NCT04389385
Extracellular vesicles (EVs)	Deliver miRNAs to inhibit inflammation and viral replication.	EVs derived from placenta MSC or placental derivatives; functional miRNAs are endogenous.	Pre	[66]
Platelet‐derived extracellular vesicles	To deliver anti‐inflammation medicine, TPCA‐1, to reduce inflammation and cytokine storm.	Targeted delivery to inflammation site in lung after intravenous injection.	Pre	[67]

^a^ Clinical stages as of January 2021.

**Pre**: preclinical study, **I**: Phase I clinical trial, **II**: Phase II clinical trial, **III**: Phase III clinical trial, **EUA**: Emergency Use Authorization by the US FDA.

## 2 NANOMATERIALS‐BASED DIAGNOSTICS FOR TRACKING SARS‐COV‐2

Diagnostic assays are critical in monitoring the spread of SARS‐CoV‐2 and enable fast identification of infected individuals and subsequent action. Currently, two conventional technologies, reverse transcription polymerase chain reaction (RT‐PCR) and computed tomography (CT) scan, are widely used in clinical practice. RT‐PCR is the most popular option for the detection of many types of viruses.^[^ ^68^ ^]^ Meanwhile, chest CT scan images may detect viral pneumonia and potential pathogenesis.^[^ ^69^ ^]^ Additionally, several other technologies contributed to the diagnostic methods. Reverse transcription‐loop mediated isothermal amplification (RT‐LAMP) assay received EUA for rapid detection of viral RNAs.^[^ ^70^ ^]^ Next generation sequencing (NGS) was initially used to confirm the identity of the novel coronavirus^[^ ^8^ ^]^ and later was combined with other technologies, such as LAMP,^[^ ^71^ ^]^ to scale up testing capacity. Microarrays using a panel of antigens or DNA probes for SARS‐CoV‐2 and/or other coronavirus were also developed to distinguish SARS‐CoV‐2 from common cold and other coronavirus diseases.^[^ ^72^, ^73^ ^]^ These techniques allow robust tests with high diagnostic sensitivity and specificity.^[^ ^74^ ^]^ However, new methods are in urgent demand for rapid testing of a large number of samples.

Nanomaterials provide useful tools for producing rapid and convenient detection methods for various pathogens.^[^ ^75^ ^]^ In particular, many gold nanoparticles (AuNPs)‐based bioassays have been developed for the detection of SARS‐CoV‐2. For example, 40 nm AuNPs were conjugated with recombinant RBD fragments of SARS‐CoV‐2 to detect anti‐RBD immunoglobulin M (IgM) and immunoglobulin G (IgG) antibodies in human serum on a lateral flow strip.^[^ ^20^ ^]^ Anti‐RBD IgG and IgM in serum were able to bind the RBD‐AuNPs conjugates, leading to gold‐based colorimetric readout visible by naked eyes. This test strip afforded 88.66% sensitivity and 90.63% specificity after analysis of 397 and 128 blood samples from RT‐PCR positive and negative patients, respectively.^[^ ^20^ ^]^ Using a similar lateral flow design, another study applied a rapid AuNPs‐based test for serum anti‐nucleocapsid IgM.^[^ ^21^ ^]^ To date, several AuNPs‐based rapid serology diagnostics have obtained EUA from the FDA.^[^ ^13^ ^]^ Besides the detection of serum antibodies, AuNPs were also used to detect SARS‐CoV‐2 antigens or viral RNAs. For instance, researchers synthesized AuNPs‐hACE2 conjugates and applied them to a microplate or cartridge surface with immobilized anti‐spike antibodies.^[^ ^22^ ^]^ In the presence of SARS‐CoV‐2, their spike proteins could be detected within 15 minutes using a microplate reader or a handheld device connected to a smartphone application.^[^ ^22^ ^]^ Similarly, in two reports, AuNPs were conjugated with antisense oligonucleotides (ASOs) complementary to the RNA sequence of SARS‐CoV‐2 nucleocapsid gene.^[^ ^23^, ^24^ ^]^ One used thio‐modified ASOs to enable a colorimetric swab test in tubes.^[^ ^23^ ^]^ The other used unlabeled ASOs for electrochemical detection on a biosensor chip.^[^ ^24^ ^]^ Viral RNAs extracted from a swab sample led to nucleic acids hybridization and agglomeration of AuNPs, which were visible to naked eyes. This assay was reported to detect as low as 0.18 ng/μL SARS‐CoV‐2 RNAs.^[^ ^23^ ^]^ In another study, gold nanoislands conjugated with DNA probes complementary to SARS‐CoV‐2 RNAs were placed on the surface of a chip sensor.^[^ ^25^ ^]^ The nucleic acid hybridization between immobilized DNAs and viral RNAs was monitored by both light (surface plasmon resonance) and heat (plasmonic photothermal effect), enabling a dual‐function detection.^[^ ^25^ ^]^ To detect COVID‐19 in exhaled breath, AuNPs were linked to an array of organic compounds that interacted with various volatile organic compounds in breath and induced change of electric resistance. Using confirmed positive and negative samples, the electric signature of COVID‐19 infection was identified by machine learning and used to detect infections.^[^ ^26^ ^]^

In addition, graphene, a thin layer of carbon atoms with high conductivity, has been widely studied as a SARS‐CoV‐2 diagnostic tool. In one study, anti‐spike antibodies immobilized on a graphene sheet were able to bind to spike proteins of SARS‐CoV‐2, resulting in changes in current and voltage, which were monitored by a field‐effect transistor.^[^ ^32^ ^]^ Based on this method, the limit of detection in nasopharyngeal swab samples of COVID‐19 patients was 242 copies of viral RNA/mL.^[^ ^32^ ^]^ Another biosensor utilized nanoflakes of reduced‐graphene‐oxide to immobilize antigens on gold electrodes.^[^ ^33^ ^]^ Anti‐SARS‐CoV‐2 antibodies in the samples bound to antigens and changed electrical impedance.^[^ ^33^ ^]^ Graphene also enabled a multiplex detection system.^[^ ^34^ ^]^ SARS‐CoV‐2 antigen and antibodies were immobilized on separate graphene electrodes to detect two antibodies (IgG and IgM), one antigen (nucleocapsid) and one inflammatory biomarker (C‐reactive protein) simultaneously in blood and saliva samples. The results provided useful information to diagnose current infection, past infection, and disease severity.^[^ ^34^ ^]^

Other materials also enabled COVID‐19 testing. One lateral flow strip used selenium NPs to detect serum IgM and IgG against the nucleoprotein of SARS‐CoV‐2 within 10 minutes.^[^ ^27^ ^]^ Lanthanide‐doped polysterene NPs were developed for fluorescence‐based lateral flow strip for serum anti‐nucleocapsid IgG.^[^ ^28^ ^]^ The accumulation of NPs loaded with europium at the test line was visualized by a portable fluorescence reader. Likewise, peroxidase‐mimic Co‐Fe@hemin nanozyme was used for the immunodetection of the RBD segment of the SARS‐CoV‐2 spike on a lateral flow test strip.^[^ ^29^ ^]^ In another report, streptavidin‐dye‐coated polymer NPs were used to facilitate the naked‐eye detection of reverse‐transcribed viral cDNA on a lateral flow strip within 1 hour.^[^ ^30^ ^]^ This method yielded more positive results than RT‐qPCR when testing 65 clinical samples obtained from feces, nasal, pharyngeal, and anal swabs. This method detected as few as five copies of the target sequence per sample.^[^ ^30^ ^]^ Cobalt‐functionalized TiO_2_ nanotubes were also reported to support the detection of the RBD surface antigen of SARS‐CoV‐2.^[^ ^31^ ^]^ This method detected the change of electrochemical signal upon RBD binding to the nanotubes without using specific antibodies.^[^ ^31^ ^]^

Magnetic NPs‐based immunoassay was also explored for testing for SARS‐CoV‐2. In one report, a filtration column with spike protein coating retained specific antibodies in the serum, which were subsequently recognized by magnetic NPs‐labeled secondary antibodies.^[^ ^35^ ^]^ The signal of accumulated magnetic NPs in the column was visualized by a portable magnetic reader. This method showed a comparable detection limit as ELISA with a shorter assay time.^[^ ^35^ ^]^ Another study reported the development of NPs with a magnetic core and a gold plasmonic shell.^[^ ^36^ ^]^ Using such magneto‐plasmonic nanoparticles, a portable device was constructed to conduct rapid RT‐PCR amplification and fluorescence detection of viral RNAs from multiple samples within 17 minutes. The detection limit was comparable to standard RT‐PCR.^[^ ^36^ ^]^

Additionally, a DNA‐based nanoswitch detected SARS‐CoV‐2 RNA by gel mobility shift induced by hybridization with fragmented viral RNA.^[^ ^39^ ^]^ Apart from independent tests, nanomaterials also aided other technologies to improve the sensitivity and specificity of other SARS‐CoV‐2 diagnostics. For example, compared with RT‐LAMP alone, the combination of crimsoned red encapsulated polymer NPs and RT‐LAMP reduced the rate of false results and shortened testing time.^[^ ^37^ ^]^

## 3 NANOMATERIALS‐BASED VACCINES FOR PREVENTING SARS‐COV‐2 INFECTIONS

Vaccines have become an important solution to control and eradicate the spread of many pathogens.^[^ ^76^ ^]^ Currently, researchers have applied numerous strategies to develop COVID‐19 vaccines.^[^ ^77^ ^]^ For example, live‐attenuated vaccines utilize engineered virus strains with reduced virulence or deoptimized codons.^[^ ^78^ ^]^ Inactivated vaccines are made from cultured whole viruses treated with sterilizing agents, such as β‐propiolactone.^[^ ^79^, ^80^ ^]^ Protein‐based vaccines use the recombinant full‐length spike protein or the RBD fragment produced by synthetic methods or living organisms.^[^ ^81^ ^]^ Nucleic acids‐based vaccines express these antigens from either DNA or mRNA.^[^ ^82^, ^83^ ^]^ Viral vector vaccines present the full‐length spike protein on the vector surface.^[^ ^84^, ^85^ ^]^ Some of these approaches have received authorization for human use, while others are under different stages of clinical trials.^[^ ^77^ ^]^

Nanomaterials are able to function as delivery systems for antigens and adjuvants.^[^ ^86^, ^87^, ^88^ ^]^ Specifically, researchers applied lipid or lipid‐derived nanoparticles (LNPs) to deliver nucleic acids‐based vaccines against COVID‐19 due to their high delivery efficiency and scalability.^[^ ^89^, ^90^, ^91^ ^]^ To date, two LNPs‐mRNA‐based vaccines, BNT162b2^[^ ^40^ ^]^ and mRNA‐1273,^[^ ^41^ ^]^ have obtained authorization from regulatory agencies in many countries and regions, such as the US FDA and European Medicines Agency. Both vaccines utilize an mRNA encoding a full‐length spike protein stabilized at the prefusion conformation by two proline substitutions (K986P and V987P). Each 0.3 mL dose of BNT162b2 contains 30 μg mRNA, 0.43 mg lipids [((4‐hydroxybutyl)azanediyl) bis(hexane‐6,1‐diyl)bis(2‐hexyldecanoate)], 0.05 mg 2[(polyethylene glycol)‐2000]‐*N,N*‐ditetradecylacetamide, 0.09 mg 1,2‐distearoyl‐sn‐glycero‐3‐phosphocholine (DSPC), and 0.2 mg cholesterol, 6 mg sucrose, and a few sodium and potassium salts.^[^ ^92^ ^]^ Each 0.5 mL dose of mRNA‐1273 encapsulates 100 μg mRNA with an ionizable LNP formulation consisting of SM‐102, polyethylene glycol 2000 dimyristoyl glycerol (PEG‐2000‐DMG), DSPC, cholesterol, and sucrose.^[^ ^93^ ^]^ Three other mRNA‐based vaccines using various ionizable LNP formulations are being evaluated in clinical trials.^[^ ^42^, ^43^, ^44^ ^]^ The mRNA in CVnCoV^[^ ^42^ ^]^ encoded the full‐length spike protein with the same two proline substitutions. Notably, MRT5500 incorporated additional mutations at the furin cleavage site to enhance prefusion stabilization.^[^ ^44^ ^]^ ARCoV utilized an mRNA that encoded the RBD fragment.^[^ ^43^ ^]^ Another vaccine candidate in preclinical studies used *N,N,N*‐tris(2‐aminoethyl) benzene‐1,3,5‐tricarboxamide derived TT3 LNPs^[^ ^94^ ^]^ to deliver mRNAs with engineered untranslated regions (UTRs), termed as NASAR to enhance antigen expression.^[^ ^45^ ^]^ The TT3 LNPs induced over 300‐fold more antigen‐specific antibodies in mice than the MC3 LNPs formulation when delivering the same NASAR mRNA encoding the full‐length spike protein.^[^ ^45^ ^]^ Additionally, researchers formulated a three‐mRNA cocktail encoding the spike, membrane, and envelope proteins of SARS‐CoV‐2 with MC3 LNPs.^[^ ^51^ ^]^ Intramuscular injection of this formulation led to the secretion of virus‐like particles and induced higher neutralizing antibody titer than the spike‐encoding mRNA alone at 4‐week time point. Meanwhile, LNPs that encapsulated self‐amplifying mRNA (SA‐mRNA) encoding a prefusion stabilized spike protein were also reported as vaccine candidates.^[^ ^47^, ^48^ ^]^ For example, LNP‐nCoVsaRNA was able to induce obvious neutralizing antibody titer in vivo and a Phase I clinical trial (ISRCTN17072692) was launched in the United Kingdom.^[^ ^95^ ^]^

Furthermore, liposomes have been studied in vaccine applications against SARS‐CoV‐2. In one study, liposomes codelivered a recombinant trimeric spike with an adjuvant, cyclic guanosine monophosphate–adenosine monophosphate (cGAMP).^[^ ^49^ ^]^ After a single intranasal inoculation, the liposome‐based vaccine elicited effective production of neutralizing antibody as well as mucosal IgA secretion.^[^ ^49^ ^]^ In another study, researchers formulated liposomes containing cobaltporphyrin‐phospholipid to convert recombinant RBD into a particulate vaccine.^[^ ^50^ ^]^ Intramuscular immunization with QS‐21, an adjuvant, in mice and rabbits mobilized cellular immunity and induced neutralizing antibodies that inhibited both pseudoviruses and SARS‐CoV‐2 infection in cell lines.^[^ ^50^ ^]^

Additionally, oil‐in‐water nanoemulsion can function as both delivery vehicles and adjuvants in vaccine development. For vaccine delivery, a squalene‐based cationic nanoemulsion delivered a SA‐mRNA encoding the spike protein.^[^ ^51^ ^]^ Upon intramuscular injection to young mice, aged mice, and pigtail macaques, the vaccine afforded antigen‐specific responses comparable to those in convalescent sera of confirmed COVID‐19 patients. For adjuvant activity, a saponin‐based nanoemulsion called Matrix M1 was used as an adjuvant in a protein‐based vaccine (NVX‐CoV2373) consisting of a recombinant trimeric spike.^[^ ^52^, ^96^ ^]^ In the Phase I/II clinical trial (NCT04368988), a prime and boost vaccination of the recombinant spike and Matrix‐M1 induced anti‐spike‐specific antibodies and the neutralizing titer was comparable to those of symptomatic patients.^[^ ^52^ ^]^ A Phase III clinical trial (NCT04583995) of this vaccine started in October 2020. Similarly, MF59, a squalene‐based nanoemulsion adjuvant, was combined with an engineered recombinant spike protein vaccine against SARS‐CoV‐2. The Phase I clinical trial of this vaccine (V451) completed in December 2020 in Australia.^[^ ^53^ ^]^ Moreover, a nanoemulsion adjuvant was recently developed by packing aluminum hydroxide (alum) on the squalene/water interphase, forming a particulate alum‐stabilized pickering emulsion (PAPE).^[^ ^54^ ^]^ Compared with alum, PAPE not only adsorbed large quantities of recombinant RBD antigens, but also enhanced dendritic cell uptake and cross‐presentation of the delivered antigens, thus increasing antigen‐specific responses in mice.^[^ ^54^ ^]^

Self‐assembled protein NPs were also explored as carriers for SARS‐CoV‐2 antigens. These protein‐based vaccines have the potential to be produced in the existing manufacturing facilities for recombinant proteins, making these vaccine candidates useful alternatives to other vaccine types. For example, I53‐50, a scaffold containing a pentameric core and a trimeric adaptor, was genetically fused with prefusion stabilized RBD of SARS‐CoV‐2.^[^ ^55^ ^]^ Upon mixing, 12 cores and 20 trimeric adaptors were self‐assembled into one nanoparticle, which carried 60 copies of RBDs. This vaccine was injected into mice together with AddaVax, a nanoemulsion adjuvant similar to MF59, and induced higher neutralizing antibodies and B cell responses than soluble recombinant RBDs. In another report, the I53‐50 scaffold presented 20 stabilized spike protein on each nanoparticle.^[^ ^56^ ^]^ Three injections of this vaccine stimulated both cellular and humoral immunities in monkeys. In subsequent SARS‐CoV‐2 challenge, the vaccinated monkeys showed reduced viral load and disease severity.^[^ ^56^ ^]^ Another self‐assembled protein vaccine was developed using the SpyTag/SpyCatcher system.^[^ ^58^ ^]^ The SpyTag was genetically fused with the RBD or the conserved heptad repeat (HR) domain of the spike protein. The SpyCatcher was genetically fused with the Helicobacter pylori ferritin, the core of the nanoparticle. Rhesus macaques inoculated by this nanoparticle generated obvious neutralizing antibodies as well as T and B cell responses. Likewise, based on the SpyTag/SpyCatcher platform, two other self‐assembled protein vaccines were also reported.^[^ ^57^, ^59^ ^]^ Recently, distinct RBDs from different human and bat coronaviruses, including SARS‐CoV and SARS‐CoV‐2, were presented simultaneously on virus‐like particle cores using the SpyTag/SpyCatcher system.^[^ ^60^ ^]^ Such a mosaic nanoparticle‐based vaccine induced cross‐reactive binding and neutralization antibodies in mice.^[^ ^60^ ^]^

## 4 NANOMATERIALS‐BASED THERAPEUTICS FOR TREATING COVID‐19

The World Health Organization (WHO) has released the therapeutic guideline for handling COVID‐19, including respiratory support, antibiotics for secondary bacterial infection, and acute respiratory distress syndrome management.^[^ ^97^ ^]^ Meanwhile, a large number of potential therapeutics for COVID‐19 are being explored in preclinical and clinical studies, such as antiviral drugs, neutralizing antibodies, immunomodulators, convalescent plasma, and cell‐based therapies.^[^ ^98^ ^]^ Currently, a few treatments have obtained governmental approvals. For example, Remdesivir, an antiviral drug, shortened the recovery time of COVID‐19 patients and received approval from the US FDA.^[^ ^99^, ^100^ ^]^ Dexamethasone, an anti‐inflammatory and immunosuppressant corticosteroid, reduced the mortality rate of severe COVID‐19 patients by one‐third, and thus obtained approval in the United Kingdom.^[^ ^101^ ^]^ Convalescent plasma with high antibody titer reduced mortality of hospitalized COVID‐19 patients in a large clinical trial and received EUA from the US FDA.^[^ ^102^ ^]^ Numerous clinical trials are ongoing to assess a wide variety of potential therapeutic agents.^[^ ^103^, ^104^ ^]^

Nanomaterials have been utilized for therapeutic applications for decades.^[^ ^105^, ^106^ ^]^ Nanomaterials can not only protect encapsulated drugs, increase intracellular delivery, and improve biodistribution, but can also function as independent therapeutics.^[^ ^88^, ^107^ ^]^ To date, multiple nanomaterials such as lipids, polymers, and cell‐derived exosomes are under development as therapeutic strategies against COVID‐19. For instance, researchers encapsulated catalase with a thin shell of polymer, improving stability and half‐life of the enzyme that degraded reactive oxygen species (ROS).^[^ ^61^ ^]^ Nebulizations and intravenous injections of the nanocapsulted catalase repressed cytokines and SARS‐CoV‐2 replication in mice and monkeys.^[^ ^61^ ^]^ Similarly, a long‐lasting polymer‐coated nanoparticulate DNase‐1 enzyme was developed to degrade large amounts of cell‐free DNA observed in the cytokine storm and sepsis induced by COVID‐19.^[^ ^62^ ^]^ Another group utilized the FDA‐approved MC3 LNPs to deliver an mRNA encoding a secreted human ACE2 receptor to inhibit the SARS‐CoV‐2 infection.^[^ ^63^ ^]^ The secreted ACE2 prevented hACE2‐expressing 293T cells from being transduced by viral particles pseudotyped with the spike protein in vitro. Additionally, LNPs were explored for the delivery of DNA or mRNA encoding neutralizing antibodies to treat COVID‐19.^[^ ^108^ ^]^

As an alternative, nanoparticles coated with ACE2‐expressing cell membrane were tested to protect healthy cells by neutralizing pseudoviruses. In one preclinical study, nanosponges were prepared by coating the cell membrane from human lung epithelial cells and macrophages onto poly(lactic‐co‐glycolic acid) nanoparticles.^[^ ^64^ ^]^ These nanosponges reduced SARS‐CoV‐2 infectivity by presenting many cell entry receptors as decoys. Along with the nanosponges, mesoporous silica‐bismuth nanorods coated with the membrane of hACE2‐expressing 293T cells were able to inhibit the invasion of pseudoviruses.^[^ ^65^ ^]^ Most recently, a nanodecoy was developed to not only neutralize the virus, but to adsorb inflammatory cytokines, such as IL‐6. The nanodecoy consisted of both hACE2‐expressing 293T cell membrane and IL‐6 expressing THP‐1 cell membrane. Based on the dual membrane assembly, the nanodecoy may provide a synergistic therapeutic effect by decreasing SARS‐CoV‐2 infectivity and mitigating lung inflammation.^[^ ^109^ ^]^

Exosomes are nanosized membrane‐bound vesicles containing many bioactive molecules, such as proteins and RNAs. A small nonrandomized primary safety clinical trial used exosomes derived from bone marrow mesenchymal stem cells (MSC), named ExoFlo, to treat 24 hospitalized COVID‐19 patients and obtained positive results.^[^ ^18^ ^]^ A randomized Phase II clinical trial of ExoFlo (NCT04493242) is ongoing. In addition, a Phase I trial (NCT04276987) examined the therapeutic effects of an aerosol inhalation of allogenic adipose MSC‐derived exosomes on severe COVID‐19 patients. Meanwhile, placental MSC‐derived extracellular vesicles were applied in delivering endogenous microRNAs as a potential therapy.^[^ ^66^ ^]^ The encapsulated microRNAs inhibited SARS‐CoV‐2 replication by targeting the viral 3′ UTR, and repressed many pro‐inflammatory cytokines, such as IL‐1β, IL‐6, and TNF‐α, in cultured cells. In another study, researchers developed platelet‐derived extracellular vesicles for treating cytokine storm through intravenous administration of these vesicles to the pulmonary inflammation sites.^[^ ^67^ ^]^ Loaded with anti‐inflammation medicine, TPCA‐1, these engineered extracellular vesicles reduced inflammation and cytokine storm in mice with pneumonia.^[^ ^67^ ^]^ Furthermore, T‐cell‐derived exosomes were explored to treat early‐stage COVID‐19 patients in an ongoing Phase I trial (NCT04389385). These exosomes were secreted from T cells stimulated by SARS‐CoV‐2 peptides in the presence of cytokines. The cellular factors inside the exosomes, such as interferon‐gamma, may benefit patients by controlling disease progression.

## 5 CONCLUSION

Decades of efforts in the field of nanomedicine have enabled researchers to adapt invaluable knowledge and experience to the design and development of diagnostics, vaccines, and treatments for COVID‐19. For COVID‐19 diagnosis, rapid testing methods that rival or exceed RT‐PCR sensitivity are still urgently needed. Different testing methods may be designed to fit specific situations.^[^ ^110^ ^]^ For example, diagnostic examination, surveillance testing, and entry screening may require different scalability and specificity based on particular purposes. Regarding vaccine development, both mRNA‐1273 and BNT162b2, two LNPs‐mRNA‐based vaccines, have entered the massive vaccination stage in many countries. Importantly, researchers need to keep evaluating and improving the current vaccines for emerging SARS‐CoV‐2 variants. According to several recent reports, some variants, such as B.1.1.7 and B.1.351, showed various degrees of resistance to the antibodies induced by the BNT162b2 and mRNA‐1273 vaccines.^[^ ^111^, ^120^, ^121^, ^122^ ^]^ Such resistance might be mediated by key mutations, like E484K in the spike protein.^[^ ^120^ ^]^ To date, no nanomaterials‐based therapeutics have yet received approval or EUA from the FDA to treat COVID‐19. Nevertheless, nanomaterials may facilitate the repurposing of current drugs in the foreseeable future. For example, dexamethasone is currently used to treat severe COVID‐19 patients.^[^ ^112^ ^]^ Prior to this pandemic, nanoformulated dexamethasone showed improved efficacy than the unformulated version against several inflammatory diseases, mostly due to the enhanced accumulation in macrophages.^[^ ^113^, ^114^, ^115^, ^116^ ^]^ Given the critical role of macrophages involved in severe COVID‐19 cases,^[^ ^117^, ^118^ ^]^ dexamethasone nanomedicine may potentially improve drug targeting to macrophages and enhance its efficacy against COVID‐19.^[^ ^119^ ^]^

Overall, advances in nanomaterials provide powerful tools for rapidly counteracting the current global public health threat. This overview of nanomaterials‐based strategies lays out a blueprint for the development of innovative and effective diagnostics, vaccines, and therapeutics for COVID‐19. These strategies can also be quickly implemented for emerging pathogens in the future.

## CONFLICT OF INTEREST

The authors declare no conflict of interest.
